# Syntheses and applications of furanyl-functionalised 2,2’:6’,2’’-terpyridines

**DOI:** 10.3762/bjoc.8.41

**Published:** 2012-03-12

**Authors:** Jérôme Husson, Michael Knorr

**Affiliations:** 1 Institut UTINAM UMR CNRS 6213, Université de Franche-Comté, Faculté des Sciences et des Techniques, 16 Route de Gray, 25030 Besançon, France; Tel.: +33-3-81676291, Fax: +33-3-81676738

**Keywords:** chelate complexes, furan, heterocycles, oligopyridines, terpyridine

## Abstract

Different synthetic routes leading to terpyridines functionalised with furan heterocycles are reviewed. The methodologies used to prepare such compounds include the ring closure of 1,5-diketones and cross-coupling reactions. These versatile terpyridines and their derived metal complexes find applications in various fields including coordination chemistry, medicinal chemistry and material sciences.

## Introduction

**Figure 1:**
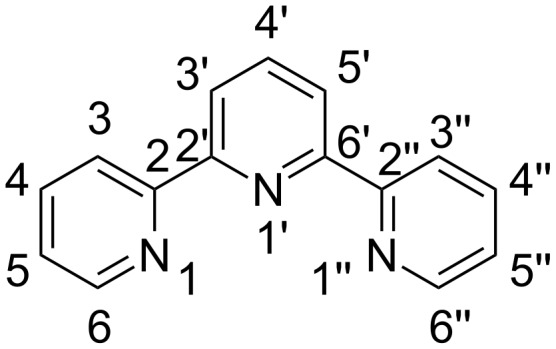
Structure and atomic numbering of 2,2’:6’,2’’-terpyridines.

Since their discovery [1–2] in the 1930s, 2,2’:6’,2’’-terpyridines (tpy) (Figure 1) have attracted widespread attention because of their excellent complexing properties as N-donor ligands toward numerous main-group, transition-metal and lanthanide cations [3].

Coordination compounds L*_n_*M(tpy)*_m_* (*n* = 0–4; *m* = 1,2) ligated with terpyridine derivatives form stable assemblies due to the thermodynamic chelate effect. In the case of transition metal complexes, the σ-donor/π-acceptor character of the dative M–N_pyridine_ bond contributes additionally to the stability of the resulting complexes. As a consequence, an impressive number of complexes have been prepared by varying the nature of the metal and by introducing various substituents onto the tpy part. A survey of the Cambridge Structural Database reveals that more than 600 complexes have been structurally characterised, with the number rapidly growing. Because of all these interesting features and properties, these compounds find widespread applications in biomedical sciences [4–5], for photovoltaic applications [6–7], as catalysts [8], etc.

On the other hand, five-membered heterocycles such as furan, pyrrole, selenophene, tellurophene or thiophene possess interesting features such as the capability to undergo chemical and electrochemical oxidation to afford polymers. These polymeric materials generally exhibit photophysical properties, making them interesting in materials science [9–12]. Finally, the rich chemistry associated with five-membered heterocycles easily allows various chemical modifications. In this respect, the attachment of such heterocycles, directly or through a linker, to a tpy system appears very interesting, since combining the intrinsic properties of the two heteroaromatics should allow both the preparation of original molecular compounds and the conception of advanced (polymeric) materials featuring novel properties. We have recently reviewed this concept for thienyl-functionalised terpyridines [13]. In contrast to the huge number of compounds of the latter type, furan-functionalised tpys have been studied to a lesser extend. Nevertheless, we feel that the interesting chemistry and potential of their furanyl-functionalised counterparts deserves to be highlighted. This minireview describes the state of the art concerning preparation and applications of such terpyridines bearing a furanyl ring.

## Review

### Synthesis by ring closure of 1,5-diketones

**Scheme 1:**
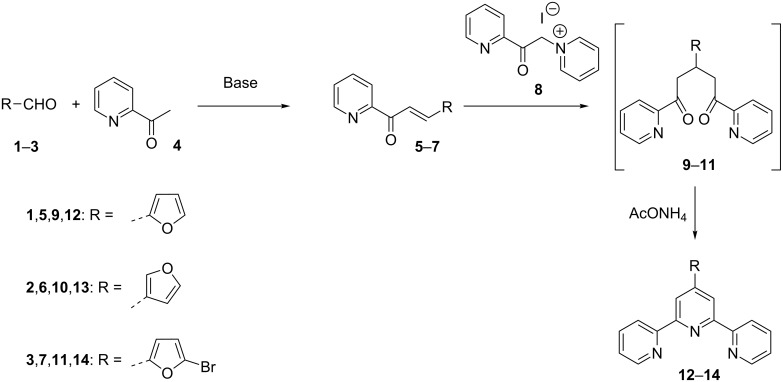
Synthesis of furanyl-substituted terpyridines **12**–**14** by using Kröhnke’s method.

In 1976, Kröhnke introduced a synthetic methodology to prepare pyridine derivatives that relies on the ring closure of 1,5-diketo-derivatives [14]. This strategy was also successfully applied to the preparation of some furanyl-substituted terpyridines. The synthetic sequence starts from furanyl aldehydes **1**–**3** and 2-acetylpyridine (**4**). The first step is a base-mediated aldol-condensation that yields the α,β unsaturated ketones **5**–**7**. Reacting these with pyridinium salt **8** afforded 1,5-diketo-derivatives **9**–**11** through Michael addition. These derivatives are generally not isolated, but undergo in situ ring closure performed in the presence of an ammonia source, such as ammonium acetate, leading to terpyridines **12**–**14** (Scheme 1) [4,15].

**Scheme 2:**
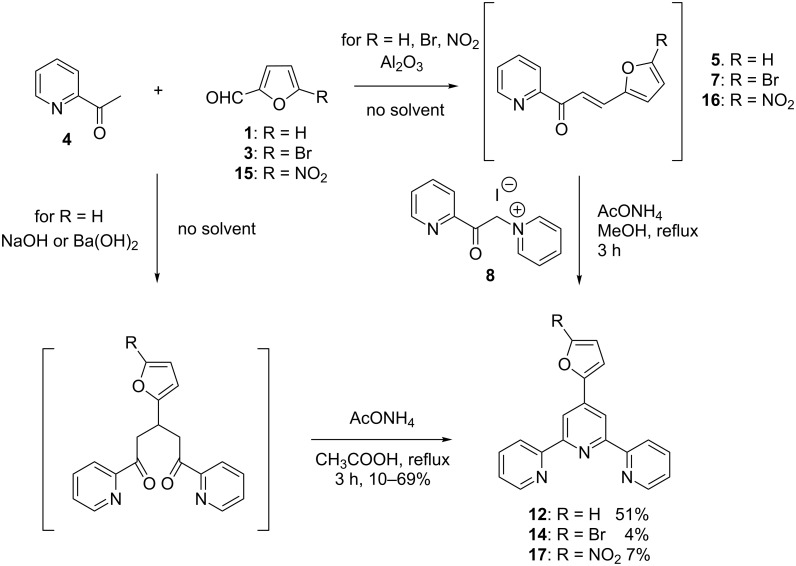
Synthesis of terpyridines under solvent-free conditions.

In the context of a more environmentally friendly and “greener” chemistry, an adaptation of this well-established method was proposed with the aim of reducing the solvent use [16–18]. Namely, two equivalents of neat 2-acetylpyridine (**4**) were reacted with one equivalent of an aldehyde in the presence of sodium hydroxide without solvent, thus yielding 1,5-diketo-derivatives. Ring closure was then carried out in methanol in the presence of ammonium acetate, according to Scheme 2. In addition to reducing the amount of solvent , this one-pot two-steps procedure avoids preparation of pyridinium salt **8**. Unfortunately, when applied to the synthesis of furanyl-substituted tpy **12**, this method leads to irreproducible results [19]. Even turning to barium hydroxide as a base (which is known to favour Michael additions [20]) did not improve the course of the reaction in a substantial manner. Therefore, basic alumina [19,21] was tested, since it is known to be an efficient promoter of aldol condensations and Michael additions under solvent-free conditions [22–23]. Nevertheless, the treatment of furanyl-substituted aldehydes **1**, **3** and **15** did not yield the targeted diketo-intermediates, but instead the chalcones **5**, **7** and **16**. The subsequent reaction of these with **8** afforded tpys **12**, **14**, and **17** in 51%, 4% and 7% yield, respectively (Scheme 2).

**Scheme 3:**
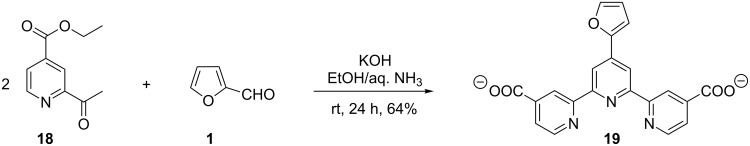
Preparation of 4,4′,4′′-trisubstituted terpyridine containing carboxylate moieties.

This “alumina” pathway not only offers better reproducibility but also allows access to tpys **14** and **17** (albeit in low yield) that could not be obtained by using sodium hydroxide or barium hydroxide. More recently, another one-pot two-steps procedure using the environmentally benign solvent ethanol was described [24]. The reaction is based on the same mechanism as the solvent-free synthesis described above, but provides better yields. This protocol was recently used to prepare the 4,4′,4′′-trisubstituted terpyridine **19** (Scheme 3), which bears two carboxylate groups at positions 4 and 4′′ and a furanyl ring on position 4′ [25]. The synthesis involves the use of ester-functionalised 2-acetylpyridine derivative **18** and furfuraldehyde (**1**). Since the reaction is performed in a basic medium, terpyridine formation is accompanied by ester hydrolysis.

**Scheme 4:**
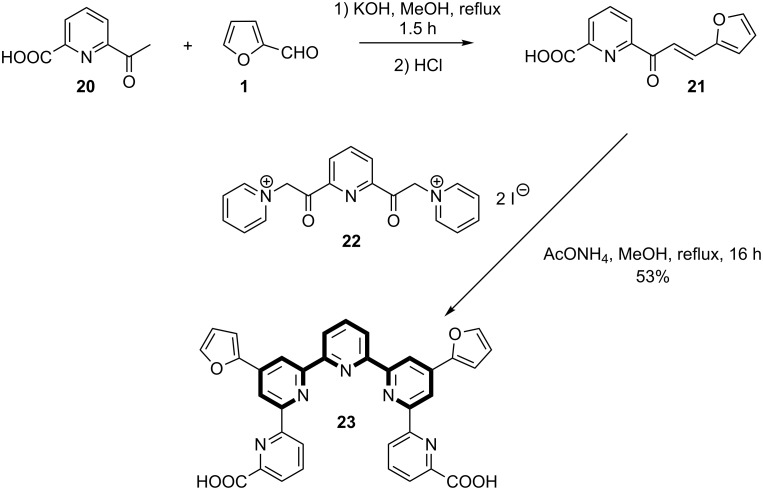
Synthetic pathway for the preparation of a furanyl-functionalised quinquepyridine.

It is interesting to note that Kröhnke’s methodology was also applied to the synthesis of a furanyl-substituted quinquepyridine, which can be considered as a terpyridine bearing two additional pyridine rings [26]. The synthesis starts with 6-carboxy-2-acetylpyridine (**20**), which is reacted with furfural (**1**), thus providing chalcone **21**. This is then reacted with di-pyridinium salt **22** in the presence of ammonium acetate to afford quinquepyridine **23** (Scheme 4).

**Scheme 5:**
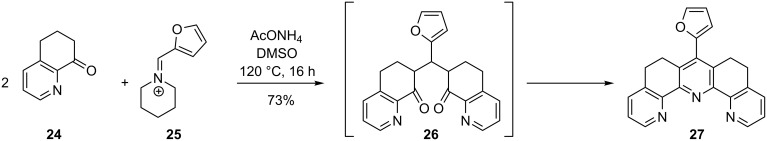
Utilization of an iminium salt in the preparation of a furanyl-substituted tpy.

Another possibility to access the key intermediate “1,5-diketone” is through the use of iminium salts as aldehyde equivalents [27]. For example, terpyridine **27** was obtained from the condensation of keto-pyridine **24** with salt **25**. The reaction proceeds via diketo-intermediate **26**, which is transformed to **27** in the presence of ammonium acetate (Scheme 5) [28].

**Figure 2:**
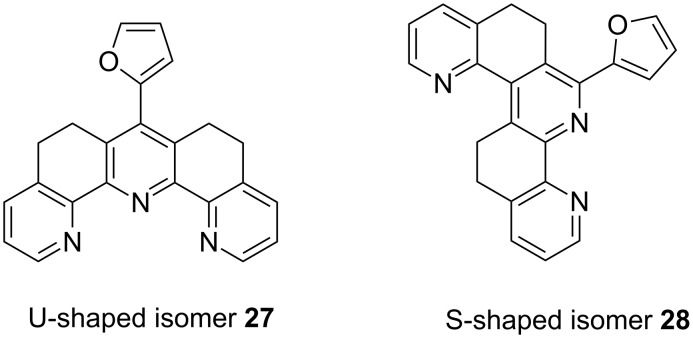
Chemical structure of U- and S-shaped isomers.

It is interesting to note that this cyclisation can lead to the formation of two different isomers [29–30], namely U- and S-shaped terpyridines **27**, **28** (Figure 2). The ratio between both isomers is solvent dependent (Table 1).

**Table 1:** Influence of solvent on U/S ratio.

Solvent	Ratio (U/S)

CH_3_CN	79/21
DMSO	100/0

**Scheme 6:**
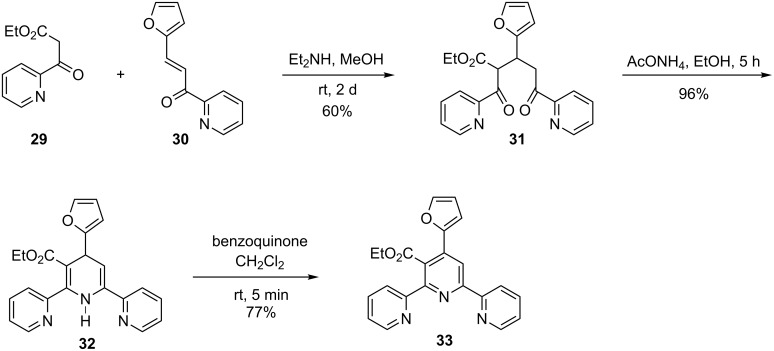
Preparation of an asymmetric furanyl-substituted terpyridine.

In all of the above-mentioned examples, symmetric terpyridines were prepared. Nevertheless, it was demonstrated that also asymmetric tpys are accessible via the 1,5-diketone pathway [31]. Michael addition of ethyl picolinoylacetate **29** to chalcone **30** affords diketone **31** in 60% yield. Reaction with ammonium acetate to effect ring closure did not yield a terpyridine, but instead dihydropyridine **32**. The latter undergoes aromatization upon reaction with benzoquinone to afford **33** (Scheme 6).

### Synthesis by cross-coupling reaction

**Scheme 7:**
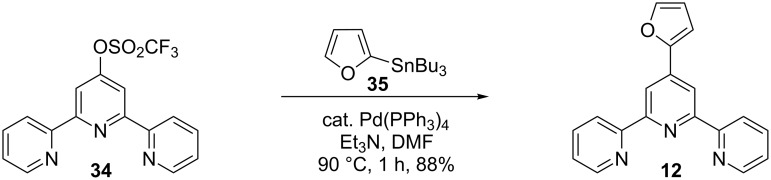
Synthesis of tpy by Stille cross-coupling reaction.

Cross-coupling reactions are widely used in organic chemistry [32] to create new C–C bonds. The importance of this technique was recently highlighted by the award of the 2010 Nobel Prize in Chemistry to Heck, Negishi and Suzuki for their contributions to the development of these reactions. Despite their widespread utilization in organic chemistry, cross-coupling reactions have been used rarely for the preparation of furanyl-substituted tpy. The only known literature example for this purpose uses the Stille reaction [33]. This C–C coupling, which involves the reaction between a halogenated or equivalent starting material and an organotin compound, was used to prepare **12** from 4′-(trifluoromethanesulfonyl)-2,2′′:6′,2′′-terpyridine (**34**) [34] and 2-tributylstannylfuran (**35**) in the presence of Pd(PPh_3_)_4_ as catalyst, according to Scheme 7 [15].

### Chemical reactivity of furanyl-functionalised tpys: preparation of carboxylate derivatives

**Scheme 8:**
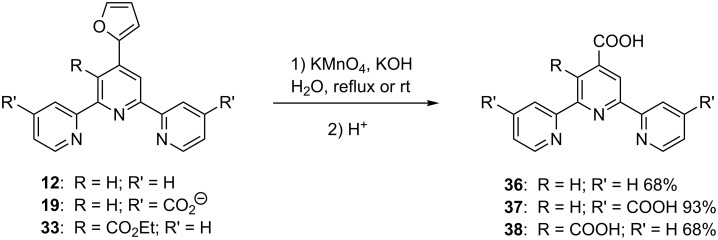
Oxidation of the furan ring of furanyl-substituted terpyridines.

The principal application of furanyl-substituted terpyridines is their use as precursors for carboxylic acid-functionalised tpys. In fact, the furan ring can readily undergo oxidative cleavage under various conditions, thus providing an interesting route to carboxylates [35]. In the case of tpys, oxidation of the furanyl ring was performed by using potassium permanganate in a basic reaction medium, followed by acidification to recover the acids. This methodology allowed the preparation of compounds **36**–**38** (Scheme 8) [25,31,36–42].

**Scheme 9:**
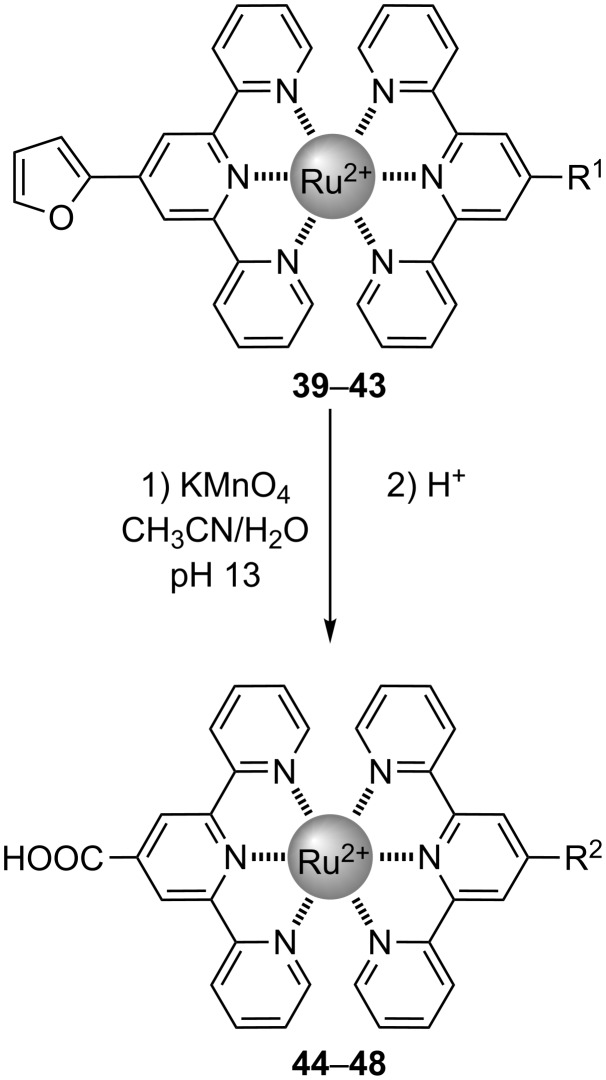
Direct oxidation of a furan ring attached on Ru(II) tpy complexes.

It is interesting to note that this procedure can be applied to the free ligand, but also directly on bis(terpyridine) Ru(II) complexes without degradation of the chelate cycles [43–47] (Scheme 9, Table 2). A series of carboxylic acid-functionalised complexes **44**–**48** was thus obtained from furanyl-functionalised complexes **39**–**43** using this methodology.

**Table 2:** Complexes obtained by direct oxidation of furanyl-functionalised tpys.

Starting materials		Final products		Yield

Compound	R^1^ =	Compound	R^2^ =	

**39**	H	**44**	H	20%
**40**	OCH_3_	**45**	OCH_3_	68%
**41**	2-thienyl	**46**	2-thienyl	51%
**42**	3-thienyl	**47**	3-thienyl	64%
**43**	2-furanyl	**48**	COOH	48%

This “furan” route to carboxylic acid functionalised terpyridines, and their corresponding complexes, presents several advantages for the preparation of tpy-COOH compared to other methods [48–50]. In particular, the starting materials are easily available, the experimental protocol is very simple, yields are improved and purification is better facilitated compared to other methods.

**Figure 3:**
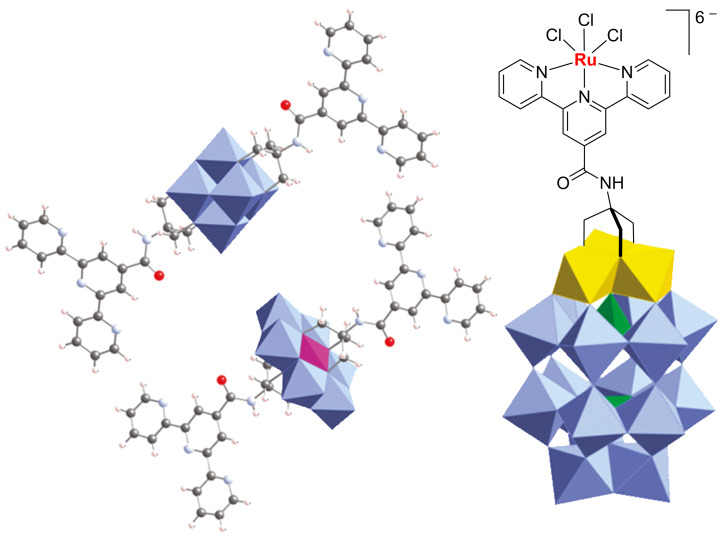
Example of polyoxometalate frameworks functionalised with tpy ligands and tpy-complex (reprinted with permission from [41], copyright (2011) American Chemical Society).

The importance of the “furyl” route to carboxylic acid-functionalised tpys and tpys-complexes is highlighted by the fact that such compounds have found applications in various domains. For instance, tpy-based materials whose synthesis includes oxidation of a furan ring during their preparation, have been used as light-harvesting materials [36,42,45–47,51–52], as chemosensors [37], in supramolecular assemblies [38–39,44], as a photocatalyst for H_2_ generation [40,53] or for the preparation of hybrid materials, such as functionalised polyoxometalates as depicted in Figure 3 [41].

### Utilization of furanyl-substituted terpyridines in biomedical sciences

Furanyl-terpyridines were probed in biomedical sciences as cytotoxic molecules. Compounds **12** and **13** were tested as anticancer agents against seven different cell lines [4]. Their activities were compared to that of doxorubicin, which is a currently used anticancer agent. Additionally, their cytotoxicity against normal cells was evaluated (Table 3).

**Table 3:** GI_50_ (μg/ml) for terpyridines **12** and **13** compared to doxorubicin. Cytotoxicities higher than the reference are highlighted in bold font.

Cell line	Doxorubicin	**12**	**13**

A-498 (Human kidney carcinoma)	6.4 × 10^−3^	**6.4 × 10^−5^**	**3.2 × 10^−5^**
PC-3 (Human prostate adenocarcinoma)	2.9 × 10^−2^	**2.5 × 10^−3^**	**4.4 × 10^−3^**
HT-29 (Human colon adenocarcinoma)	4.6 × 10^−3^	**2.4 × 10^−3^**	**2.6 × 10^−3^**
A-549 (Human lung carcinoma)	9.2 × 10^−2^	2.2 × 10^−1^	**8.0 × 10^−2^**
HCT-15 (Human colon adenocarcinoma)	7.1 × 10^−2^	1.0 × 10^−1^	**6.0 × 10^−2^**
SK-OV-3 (Human ovary adenocarcinoma)	7.3 × 10^−2^	1.3 × 10^−1^	6.0 × 10^−1^
SK-MEL-2 (Human malignant melanoma)	5.9 × 10^−2^	9.9	3.3 × 10^−1^
RPTEC (Renal proximal tubule epithelial cells)	7.2 × 10^−2^	**4.1 × 10^−3^**	**5.8 × 10^−3^**

As can be seen from Table 3, these furanyl-functionalised terpyridines display, in many cases, better cytotoxicity than doxorubicin. Unfortunately, they are also more toxic toward normal cells (RPTEC). Note that, to date, the exact molecular mechanism of physiological action for these compounds has not yet been clearly elucidated.

**Scheme 10:**
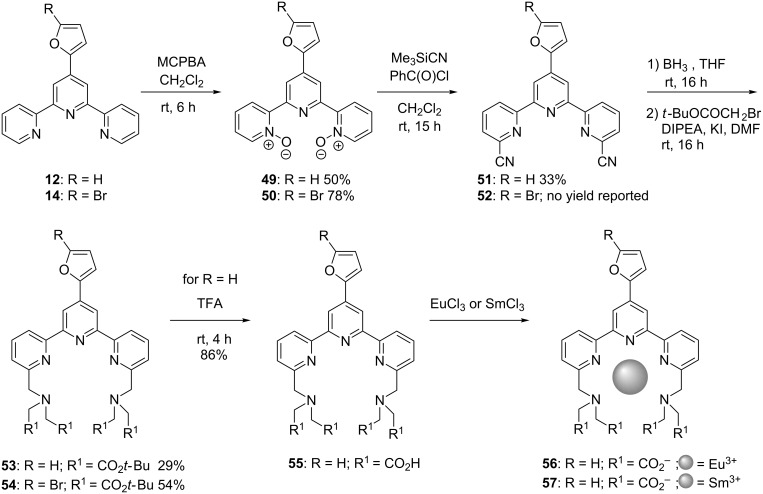
Synthetic pathway to europium(III) and samarium(III) chelates **56** and **57**.

Terpyridines **12** and **14** were also used as starting materials for the preparation of chelating agents and complexes with the aim of making fluorescent labels for biomolecules [15]. The synthetic pathway begins with the preparation of *N*,*N*′-dioxides **49** and **50** upon reaction with MCPBA. Treatment with trimethylsilyl cyanide allowed the introduction of a cyano group at the α-position with respect to the N-atoms, thus yielding the bis(nitrile) compounds **51** and **52**. The cyano group was then converted to an amine function by reduction with BH_3_. Subsequent treatment with *tert*-butyl bromoacetate afforded amino esters **53** and **54**. Ester groups of compound **53** were then hydrolyzed to the free acids to afford **55**. The latter was then reacted with europium(III) or samarium(III) chlorides to provide lanthanide complexes **56** and **57** (Scheme 10).

**Scheme 11:**
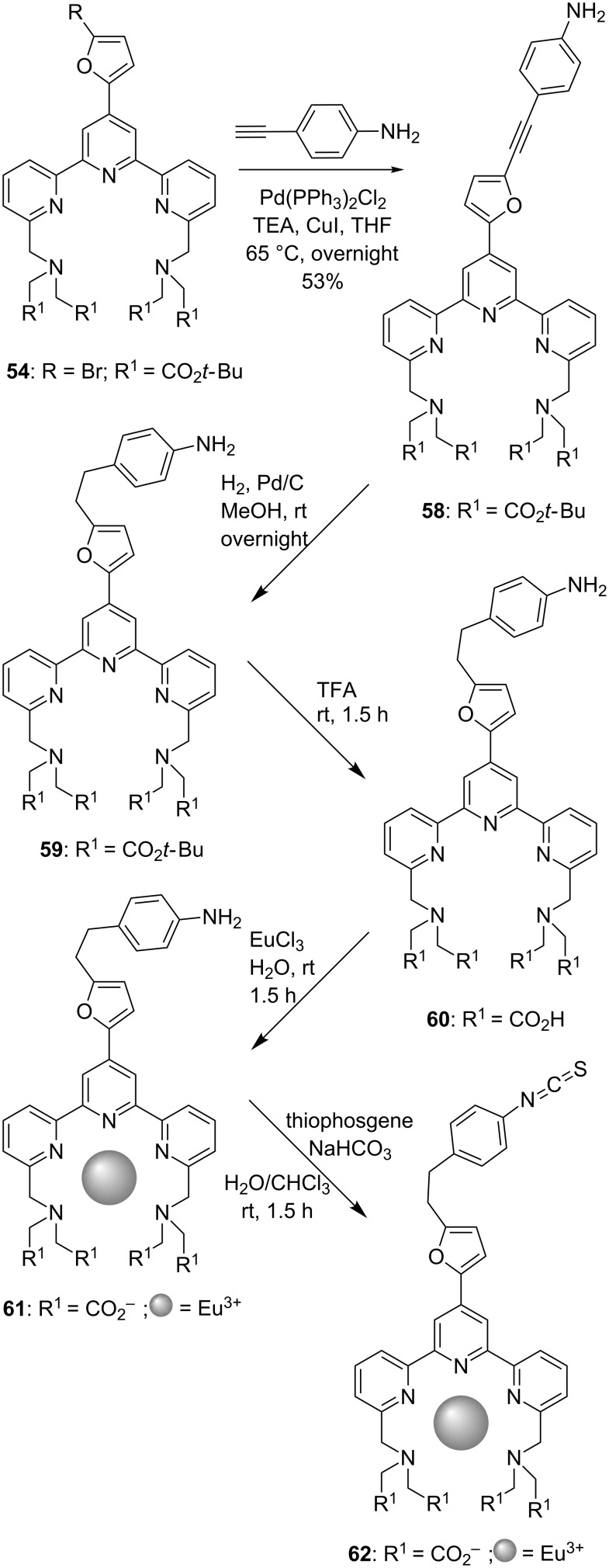
Synthetic pathway to prepare thiocyanato-functionalised tpys as potential biomolecule-labelling agents.

The bromo-derivative **54** was further functionalised by cross-coupling reactions. Namely, the treatment of **54** with aminophenylacetylene in a Sonogashira reaction afforded terpyridine **58**. The triple bond was then reduced by hydrogenation providing tpy **59**, which features an alkyl spacer between the furanyl and phenyl ring. Cleavage of the pending ester moieties yielded polycarboxylic acid compound **60**, which was reacted with EuCl_3_ leading to chelate complex **61**. Finally, nucleophilic addition of the –NH_2_ groups of **61** to thiophosgene yielded terpyridine complex **62**, which bears a thiocyanato group at the *para*-position (Scheme 11).

**Scheme 12:**
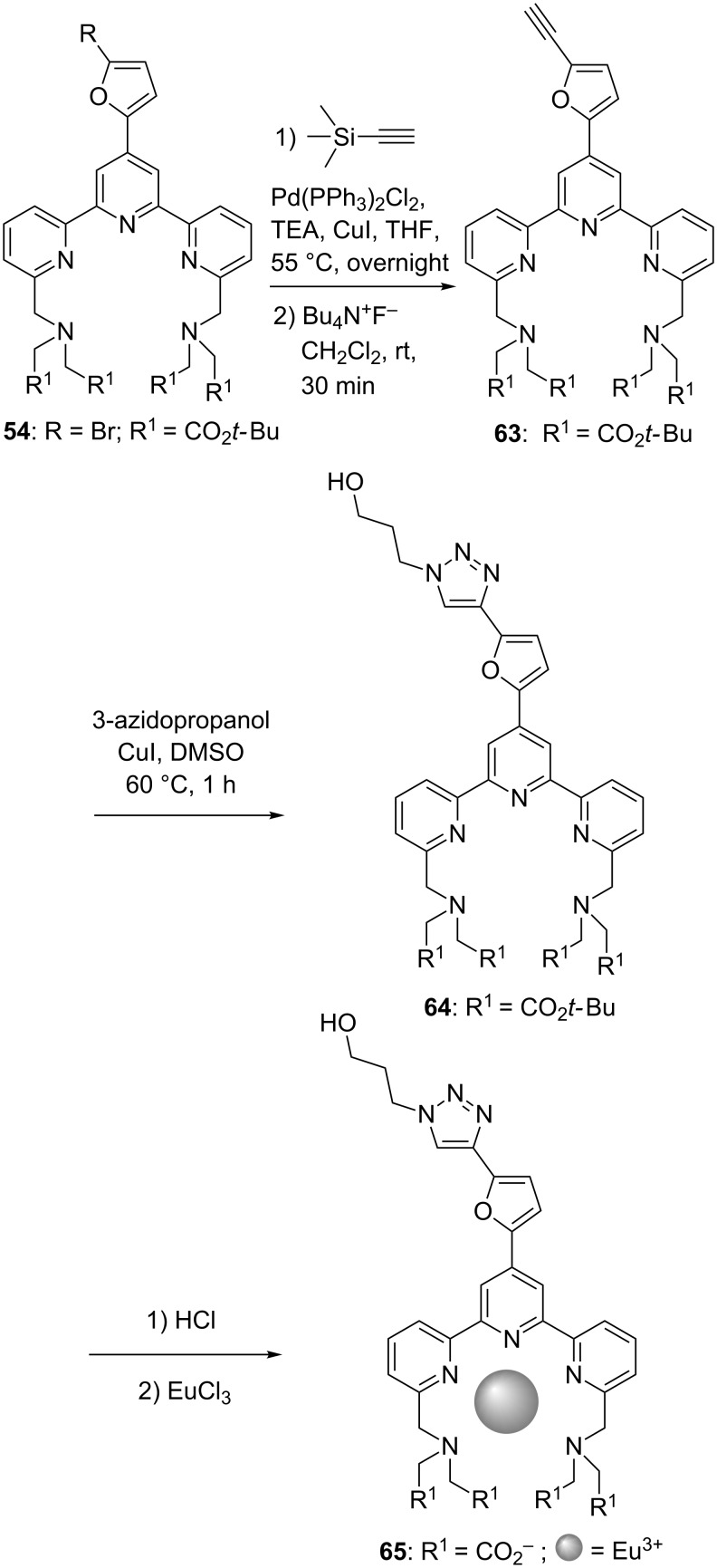
Synthetic sequence envisioned for biomolecules labelling by click-chemistry.

Because of the presence of a thiocyanato moiety in its structural motif and due to its fluorescence properties, compound **62** could be used potentially as a labelling agent for biomolecules. In fact, reaction between the reactive electrophilic thiocyanato group and a nucleophilic residue present on the biomolecule (amine, thiol, for example) should allow anchorage of **62**. Another approach that was envisioned for the linking of fluorescent europium–terpyridine complexes to biomolecules is the use of click chemistry [54]. Again, **54** was used as a starting material and reacted with trimethylsilyl acetylene in a Sonogashira reaction. After deprotection of the trimethylsilyl moiety with tetrabutylammonium fluoride, terpyridine **63** was obtained. Reaction of the latter with 3-azidopropanol afforded the triazinyl-containing compound **64**. Finally, the hydrolysis of the ester moieties followed by complexation to Eu(III) yielded the lanthanide complex **65** (Scheme 12).

This synthetic sequence was used to demonstrate the possibility to prepare labelled oligonucleotides starting from azido-functionalised ones and alkynyl-containing terpyridines. In all the examples cited above, furanyl-functionalised terpyridines were selected because of their capability to absorb light and to transfer the excitation energy to the chelated lanthanide metal ion. This gives rise to a strong fluorescence, a feature which is required for labelling studies.

### Miscellaneous utilization of furanyl-functionalised tpys

**Figure 4:**
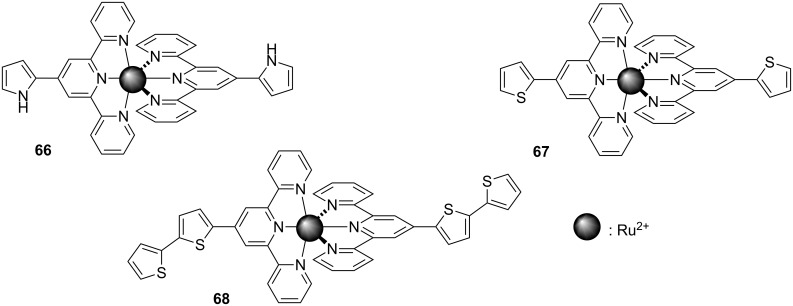
Structure of pyrrolyl (**66**), thienyl (**67**) and bithienyl (**68**)-substituted complexes analogous to compound **43**. PF_6_^−^ counter-anions are omitted.

Terpyridines functionalised with five-membered heterocycles, especially thiophene [13], and their derived complexes have proved to be interesting materials in the field of dye-sensitized solar cells (DSSC) [55]. Understanding in depth both the electrochemical and photophysical properties of such compounds is therefore of crucial importance. In this context, the octahedral Ru(II) complex **43** was studied and compared with analogous pyrrolyl, thienyl and bithienyl-substituted compounds **66**–**68** (Figure 4) in order to obtain information about the influence of the pendant heterocycle on the physicochemical properties [56].

It appeared from this study that the bithienyl substituent has the most pronounced effect on the photophysical properties of this series of Ru complexes. Especially, **68** absorbs at a longer wavelength (Table 4), which constitutes an important requirement for sensitizers to be used in DSSC [6].

**Table 4:** UV–vis and emission data for complexes **43** and **66**–**68**.

Complex	λ_max,abs_ (nm)	λ_max,em_ (nm)

**43**	500	660
**66**	507	665
**67**	499	670
**68**	514	710

## Conclusion

This short review demonstrates that the combination of the furan heterocycle and the terpyridine ligand leads to a series of quite versatile functional molecules. The attached furan ring serves mainly as a precursor to carboxylic acid moieties with the final aim of preparing functionalised materials for application in devices, especially solar cells. Recent results obtained in this field with furan-containing molecules [57–59] demonstrate that the introduction of this heterocycle onto terpyridine is an interesting strategy. Additionally, furan-functionalised terpyridines revealed a potential utility in biomedical sciences. Owing to the rich chemistry associated with furan and pyridine rings, it is realistic to imagine the use of furanyl-functionalised terpyridines as a platform for further functionalisation to elaborate even more sophisticated compounds that may be useful in diverse domains.
